# Holmium Oxide Glass Wavelength Standards

**DOI:** 10.6028/jres.112.024

**Published:** 2007-12-01

**Authors:** David W. Allen

**Affiliations:** National Institute of Standards and Technology, Gaithersburg, MD 20899-8442

**Keywords:** calibration, holmium oxide, spectrophotometer, wavelength scale

## Abstract

Holmium oxide glass has been used as a wavelength standard for over four decades. These standards have shown insignificant spectral variation from batch to batch and from one manufacturer to another. The National Institute of Standards and Technology (NIST) has certified and recertified holmium oxide glass samples for over four decades. Over this period of time there has been no recorded instance of a spectral shift of the certified bands for any of the samples measured. Moreover, these samples are known to be robust and relatively insensitive to a normal range of temperature and humidity. Based on the extensive experience that NIST has with this material and its long-term stability, NIST will no longer recommend the recertification of these standards. Furthermore, traceability may be established either through the supplier or by the end user without the need for NIST involvement.

## 1. Introduction

Holmium oxide glass is commonly used as a wavelength standard for the calibration of spectrophotometers. It has a number of desirable features that have made it a commonly used wavelength standard. It does not induce a slit positioning error as atomic emission lamps may. It is also compact, easy to use, and most importantly, stable over long periods of time.

NIST, formerly known as the National Bureau of Standards (NBS), began distributing holmium oxide glass filters in 1961 [1]. The glass was initially developed by Corning Glass, NY and sold as glass No 3130 [2]. The thickness of the glass selected was typically less than 3 mm, which provides 1 % transmittance or greater at the absorption peaks. The absorption bands were determined by consensus of several laboratories using different instruments. Eleven bands were selected based on their sharp and symmetric absorption peaks [1].

NIST has been involved in the dissemination of several different types of holmium oxide standards. These include 52 mm square glass, glass within a cuvette mount, and a liquid solution in cuvette cells. The glass samples were once offered for direct sale (calibration service 38050C and 38051C) and the solution was provided as a Standard Reference Material (SRM 2034) [3]. NIST no longer provides these samples, as they are readily available through commercial suppliers, and traceability for such measurements can be achieved as described below.

Although both the holmium oxide glass and the solution are based on the same rare earth oxide, they exhibit slightly different wavelength bands. Additionally, there are some near infrared bands in the glass that are not present in the solution due to absorption by the water. The bands discussed in this paper should only be used in conjunction with the glass standard. For the purpose of this paper, reference to holmium oxide filters refers to the glass variety and not the liquid solution.

**Fig. 1:**
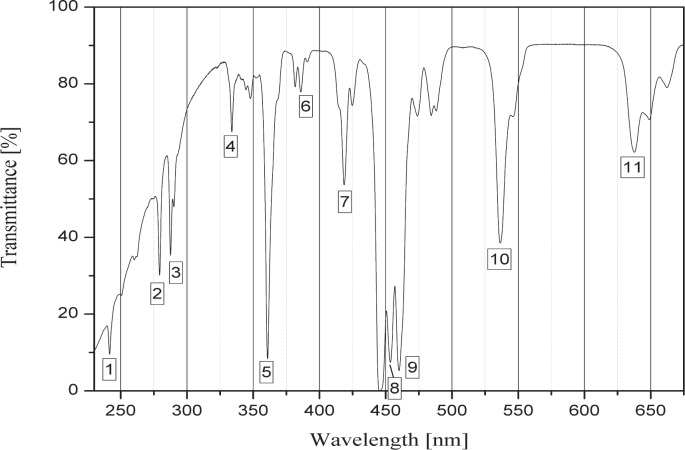
Spectral transmittance of holmium oxide glass showing 11 NIST certified bands. A line has been inserted for band 8 for clarification. The position of the wavelength minima is the critical parameter, while the level of transmittance can be considered irrelevant.

One well recognized difference observed between different batches of holmium oxide filters is the presence or absence of the 241 nm band, labeled 1 in Fig. 1. Some filters have either a less distinct absorption peak or no peak at all. This is likely due to the variation in the base glass composition causing reduced UV transmittance. Typically, the remaining bands are sufficient to serve the needs of the user.

It is important to note that while these standards are inherently stable with respect to the wavelength scale (abscissa), the transmittance scale (ordinate), also historically referred to as the photometric scale, can be subject to change due to temperature, surface contaminants and other environmental sources. Therefore, it is important that these holmium oxide standards not be used in the calibration of the transmittance scale. Typically, the transmittance scale is determined using a set of neutral density filters. For a complete set of procedures for measuring the performance of spectrophotometers see reference [4].

The following figures show the typical signature for holmium oxide glass. Although the band depth may appear different from those shown here, the band position is the key factor. The certified bands are listed in Table 1.

## 2. Traceability

The transition of the holmium oxide glass standard to one that does not need direct involvement of NIST through a calibration service parallels the transition which was described by Travis, *et al.* for the holmium oxide solution [5]. That paper discusses the bands as intrinsic values with the establishment of traceability through atomic absorption lines of the Hg lamps that were used to establish the certified bands. Those atomic lines are directly traceable to the values of NIST standards for the SI unit, the meter.

**Fig. 2:**
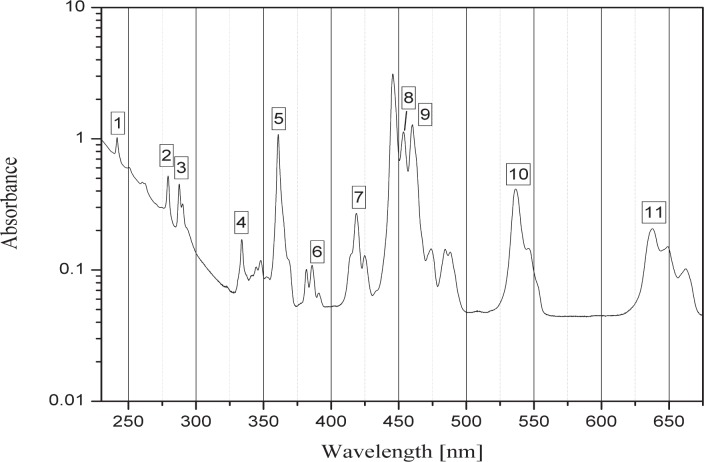
Absorbance peaks of the 11 NIST certified bands are shown for convenience. A line has been inserted for band 8 for clarification. The absorbance is a calculated quantity derived from the measured spectral transmittance.

The user of a holmium oxide filter can assess the presence of the certified bands using a reference such as another certified holmium oxide filter or, alternatively the user can overlap the spectra for the sample in question with a reference spectrum (digital copy provided by NIST). There may be some differences between the measured sample and the reference, but the presence of a contaminant would lead to a significantly different feature not present in the reference spectrum. Once the user has demonstrated that the same absorption features are present as the ones shown in Figs. 1 and 2, the user can self-declare traceability to the SI (International System of Units). Likewise, the same procedure may be followed by a supplier, which in turn may provide SI traceability for the holmium oxide filters sold.

Once a sample has been verified to be holmium oxide glass, based on the known absorption bands, there is no need to recertify the sample. Since holmium oxide is inherently more stable than most instruments, it is not meaningful to measure the sample with the intention of calibrating the sample itself. The purpose of the measurement is instead to identify the standard as a holmium oxide wavelength standard. The holmium oxide standard in essence may be considered an intrinsic standard.

The certified wavelengths of minimum transmittance are considered to be valid for spectral bandwidths not exceeding 2 nm. Spectral bandwidths greater than 2 nm can lead to errors of minimum transmittance due to asymmetry of the band. The expanded uncertainty (*k* = 2) [6] of the certified values is 0.2 nm.

## 3. Summary

Based on the extensive experience that NIST has with holmium oxide glass wavelength standards, NIST no longer recommends the recertification of the wavelength standards. Matching to the absorption bands listed in this paper provides traceability to SI through self declaration.

## Figures and Tables

**Table 1:** Certified wavelengths of minimum transmittance of the holmium oxide glass filter for a spectral bandwidth of 1 nm. Band 1 may be absent from some glasses due to UV absorbance.

Band	Certified wavelengths [nm], *k* = 2
1	241.5 + / − 0.2
2	279.3 + / − 0.2
3	287.6 + / − 0.2
4	333.8 + / − 0.2
5	360.8 + / − 0.2
6	385.8 + / − 0.2
7	418.5 + / − 0.2
8	453.4 + / − 0.2
9	459.9 + / − 0.2
10	536.4 + / − 0.2
11	637.5 + / − 0.2
